# Optimization Technology of the LHS-1 Strain for Degrading Gallnut Water Extract and Appraisal of Benzene Ring Derivatives from Fermented Gallnut Water Extract Pyrolysis by Py-GC/MS

**DOI:** 10.3390/molecules22122253

**Published:** 2017-12-20

**Authors:** Chengzhang Wang, Wenjun Li

**Affiliations:** 1 Institute of Chemical Industry of Forest Products, Chinese Academy Forestry, Nanjing 210042, China; liwenjun611@163.com; 2 National Engineering Laboratory for Biomass Chemical Utilization, Nanjing 210042, China; 3 Key and Open Laboratory on Forest Chemical Engineering, State Forestry Administration, Nanjing 210042, China; 4 Key Laboratory of Biomass Energy and Material, Nanjing 210042, China; 5 Institute of New Technology of Forestry, Chinese Academy Forestry, Beijing 100091, China

**Keywords:** gallnut water extract, tannic acid, benzene ring derivatives, biodegradation, Py-GC/MS

## Abstract

Gallnut water extract (GWE) enriches 80~90% of gallnut tannic acid (TA). In order to study the biodegradation of GWE into gallic acid (GA), the LHS-1 strain, a variant of *Aspergillus niger*, was chosen to determine the optimal degradation parameters for maximum production of GA by the response surface method. Pyrolysis–gas chromatography–mass spectrometry (Py-GC/MS) was first applied to appraise benzene ring derivatives of fermented GWE (FGWE) pyrolysis by comparison with the pyrolytic products of a tannic acid standard sample (TAS) and GWE. The results showed that optimum conditions were at 31 °C and pH of 5, with a 50-h incubation period and 0.1 g·L^−1^ of TA as substrate. The maximum yields of GA and tannase were 63~65 mg·mL^−1^ and 1.17 U·mL^−1^, respectively. Over 20 kinds of compounds were identified as linear hydrocarbons and benzene ring derivatives based on GA and glucose. The key benzene ring derivatives were 3,4,5-trimethoxybenzoic acid methyl ester, 3-methoxy-1,2-benzenediol, and 4-hydroxy-3,5-dimethoxy-benzoic acid hydrazide.

## 1. Introduction

**Figure 1:**
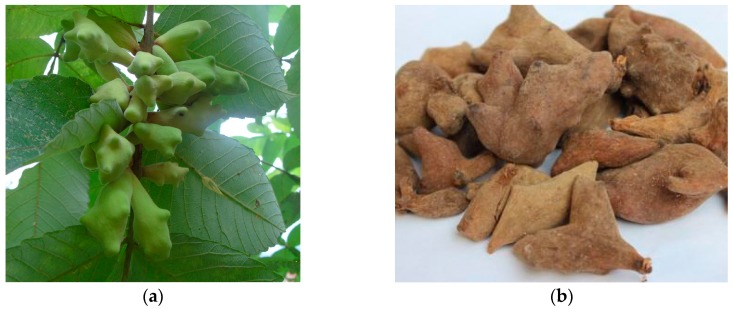
Chinese Du-ensiform gallnut, (**a**) fresh gallnut; (**b**) dry gallnut.

Gallnut is an abnormal gall in China produced by parasitic aphids on the host trees of *Rhus chinensis*, *Rhus potaninii*, or *Rhus punjabensis*, etc. Chinese gallnut can be divided into the three categories of Du-ensiform gall, horned gall, and gall flowers, according to aphid species and host differences. Du-ensiform gall enriches 55~65% of tannin (Figure 1), and is the most traditional and important raw material for producing gallnut water extract (GWE) which contains 80~90% tannin. GWE has been used to produce gallic acid through the hydrolysis of alkali, and acid catalysis. However, chemical degradation of GWE not only provides very low yields of gallic acid, but causes serious environmental pollution and equipment corrosion. Therefore, it is urgent to probe more environmentally friendly degradation technology in order to produce gallic acid and its derivatives [1,2].

The enzymatic hydrolysis of GWE using the microorganisms of *Aspergillus* and *Penicillium*, particularly *Aspergillus niger*, has great potential for the industrialized production of gallic acid [3]. Wang [4] separated one strain of *A. niger* from Chinese Gall “Beihua” and fermented gallnut directly to produce GA with a yield of 78.5~89.5%. Yang [5] used the same strain to ferment gallotannins and purified macroporous resin to obtain a high yield of 85.6% gallic acid. Banerjee modified a tannin-rich substrate of solid-state fermentation (MSSF) to produce tannase and gallic acid through co-culture method to obtain maximum yield of 94.8% gallic acid (GA) [6]. However, no literature has reported on fermented GWE chemical constitutes and structures so far, particularly with respect to the biodegradation mechanism of GWE biotransformation.

Since the microbial transformation of tannins was discovered by Tieghem [7], only a few analytical techniques, such as high-performance liquid chromatography (HPLC), thin-layer chromatography (TLC), or pyrolysis–gas chromatography–mass spectrometry (Py-GC/MS) have been applied to the identification of natural products and their metabolites by both conventional destructive or non-destructive methods in the process of chemical synthesis and biotransformation [8,9,10,11]. In order to develop gallic acid by microbial degradation of GWE, Min previously reported the extraction and screening of tannic acid degradation by the LHS-1 strain according to the indexes of tannic acid degradation rate, gallic acid accumulation concentration, and tannin enzyme activity [12]. This study focused on the optimization technology of LHS-1 strain degradation of GWE, and Py-GC/MS was applied to probe the chemical constituents and structural characterization of the linear and benzene ring derivatives, based on phenolic and glucose structure from fermented GWE (FGWE) pyrolysis. It will be very helpful for us to research the biodegradation mechanism and the corresponding metabolites of GWE for the future.

## 2. Results

### 2.1. Determination of Tannic Acid and Gallic Acid in FGWE

**Figure 2:**
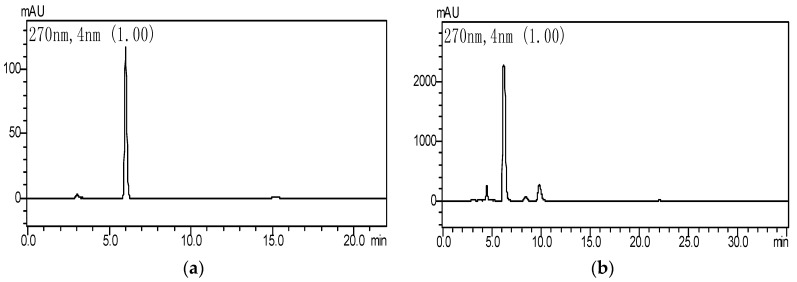
HPLC of a standard gallic acid sample and fermented gallnut water extract (FGWE), (**a**) standard sample of gallic acid; (**b**) the fermentation broth of GWE.

Folin–Ciocalteau and HPLC were both used to monitor the changes in gallic acid and tannic acid in GWE biodegradation with the LHS-1 strain. The standard curves of tannic acid and gallic acid had higher linearity. Tannic acid was detected at 630 nm, and its equation was *y* = 69.848*x* + 0.0011 (*R*^2^ = 0.9993), while the equation of gallic acid was *y* = 0.0029*x* + 0.0039 (*R*^2^ = 0.9998). The analysis showed GWE contained 81.6% of tannin acid and 5.6% of gallic acid. When GWE was incubated at 28 °C with a pH of 5.0 for 50 h, HPLC showed that the levels of tannic acid decreased and those of gallic acid increased significantly in FGWE (Figure 2).

**Figure 3:**
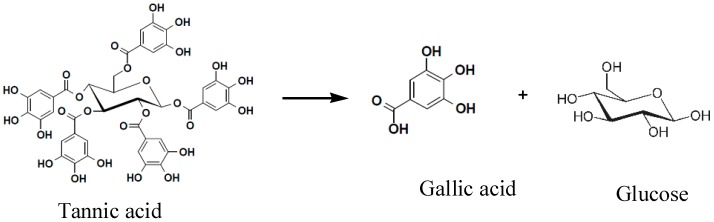
The degradation of GWE tannic acid into gallic acid.

The degradation rate of GWE tannic acid exceeded 85.6%. The concentration of gallic acid approached 63~65 mg·mL^−1^, and tannin enzyme activity was 1.17 U·mL^−1^. This indicated that the LHS-1 strain could produce tannase to transform tannic acid into gallic acid (Figure 3).

### 2.2. Box–Behnken Design and Variance Analysis of the LHS-1 Strain for Degrading Tannic Acid

Since the incubation temperature, the initial pH, and the incubation period obviously affected tannin enzyme activities in the culture media, and the ability of the LHS-1 strain to degrade tannic acid was measured through the degradation rate of tannic acid, the concentration of gallic acid, and the activity of tannase, the Box–Behnken experiment was designed and the results are shown in Table 1. The regression equation was determined as follows:

$$\begin{array}{l} Y=1.18+0.039X_{1}+0.002X_{2}+0.35X_{3}-0.003X_{1}X_{2}-0.007X_{1}X_{3}+\\ 0.045X_{2}X_{3}-0.10X_{1}{}^{2}-0.13X_{2}{}^{2}-0.15X_{3}{}^{2} \end{array}$$

Variance analysis and significant tests of this regression model are shown in Table 2. The quadratic model was very significant and meaningful in statistics (*F* = 23.62, *p* = 0.0002 < 0.001). The lack-of-fit item was good (*p* = 0.3680 > 0.05), and no loss factors existed. Therefore, the regression equation could substitute the real experiment to analyze and direct experiments. For the monomial items (*X*_1_, *X*_2_, and *X*_3_), the effects of culture temperature and incubation time on tannase activities were significantly different (*p* <0.05). The effects of the three monomial factors on tannase activities ranging from strong to weak were in the following order: incubation temperature (*X*_1_) > incubation time (*X*_2_) > incubation initial pH (*X*_3_). For interactive items of *X*_2_*X*_3_ (*p* = 0.0352 < 0.05), initial incubation pH and the incubation time showed a great interaction, while *X*_1_*X*_2_ and *X*_1_*X*_3_ had no interaction (*p* > 0.05). For the binomial items of the equation, the effects of the incubation temperature, initial pH, and incubation time on tannin enzyme activities reached significant and extreme levels (*p* < 0.0001). Through comparing the *p*-value, the primary and secondary factors could be judged for the LHS-1 strain with respect to the degradation of tannic acid [13].

### 2.3. The Optimization Technology for the LHS-1 Strain to Degrade Tannin

**Figure 4:**
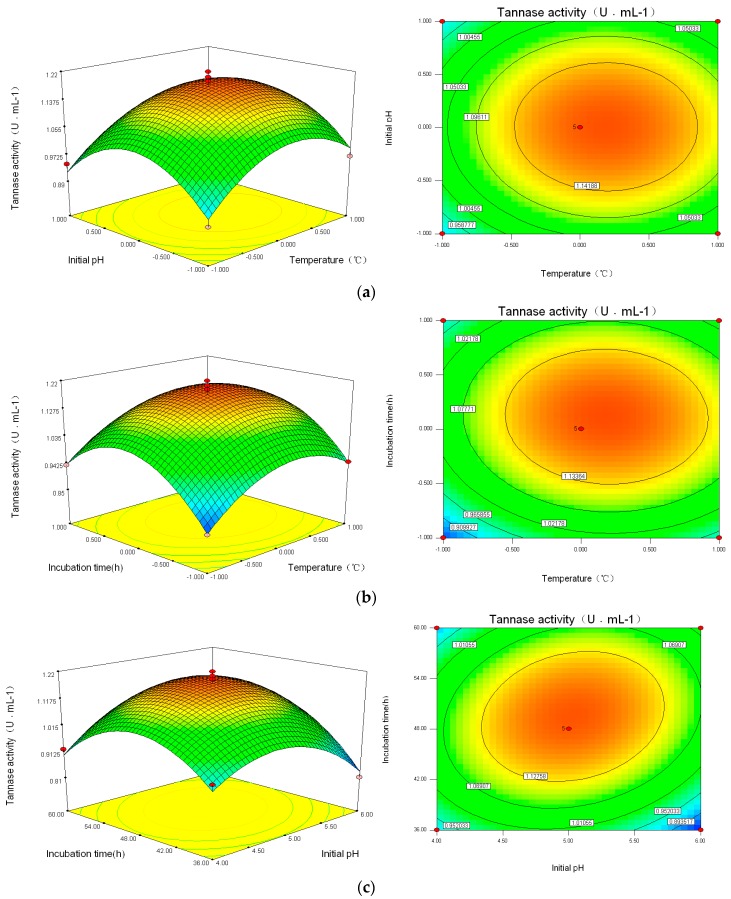
Contour plots and 3D-response surface plots showing (**a**) the interactive effects of incubation temperature and initial pH (*X*_1_*X*_2_); (**b**) incubation temperature and incubation time (*X*_1_*X*_3_); and (**c**) initial pH and incubation time (*X*_2_*X*_3_).

According to the regression analysis and Box–Behnken experimental optimal model, the contour plots and 3D response surface plots were obtained by the Design Expert software, as shown in Figure 4, which are confined in the smallest ellipse in the contour diagram. The tannin enzyme activities were obtained with two continuous variables, while the other variables were fixed constantly. Elliptical contours were obtained when there was a perfect interaction between the independent variables. For *X*_2_*X*_3_ there existed a significant interaction (*p* < 0.05) and the contour map was shown to be elliptical. However, *X*_1_*X*_2_ and *X*_1_*X*_3_ showed round contours with no significant interaction (*p* > 0.05), indicating that *X*_1_*X*_2_ and *X*_1_*X*_3_ had less of an effect on tannin enzyme activities. Hence, initial pH and incubation time had more obvious effects. The maximum tannin enzyme activities were obtained when the temperature was around 29~33 °C, with pH of 4.5~5.5, and an incubation time of 42~54 h.

In the Box–Behnken design, the optimal conditions were determined as follows: cultivating temperature 30.92 °C, initial pH 5.03, and incubation time of 49.44 h, with the corresponding tannin enzyme activity of 1.192 U·mL^−1^. Considering the practical experiments, the optimal parameters were adjusted to a temperature of 31 °C, initial pH of 5, and incubation time of 50 h, with tannin enzyme activity of 1.17 U·mL^−1^. The relative error of the model was 1.68% (*R* < 5%). Thus, the response model could reflect the expected optimization. Compared with an enzyme activity of 0.973 U·mL^−1^, the LHS-1 strain could increase the tannin enzyme activity by more 20.2% in the optimal fermentation condition.

### 2.4. The Appraisal of Linear and Benzene Ring Derivatives Based on Phenolic and Glucose Structure from FGWE Pyrolysis by Py-GC/MS

**Figure 5:**
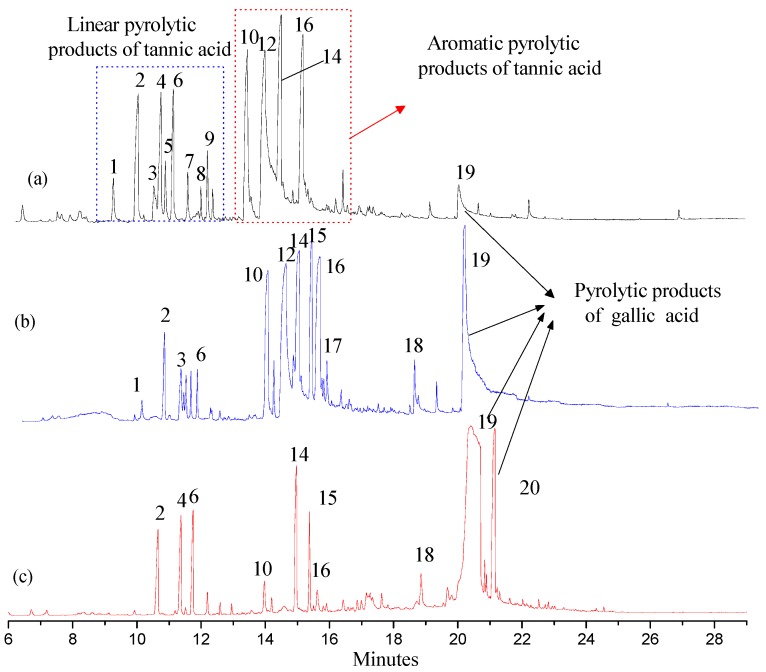
The total ion chromatography of three samples with pyrolysis–gas chromatography–mass spectrometry (Py-GC/MS). (**a**) Tannic acid standard sample (TAS); (**b**) GWE; (**c**) FGWE.

Py-GC/MS was used to appraise the pyrolytic products of FGWE pyrolysis through comparison with the tannic acid standard sample (TAS) and GWE data, combined with GC-MS to determine gallic acid and tannic acid levels in samples. Their total ion chromatography peaks all consisted of linear and aromatic products, with retention times ranging from 9 to 13 min and 13 to 16 min, respectively, as well as gallic acid structural products with retention times ranging from 16 to 21.15 min. Through comparison of the mass spectra of GC-MS retention times with corresponding standards, over 20 kinds of compounds were identified as linear and benzene ring matrix structures (Figure 5). The linear compounds, marked with numbers 1~9, were identified because of the pyrolysis of glucose, and benzene ring compounds were marked with numbers 10~20, which were appraised as the results of pyrolysis of ester bonds between gallic acid and glucose and epside bonds between gallic acids.

After pyrolysis, TAS produced 9 linear compounds and 5 benzene ring derivatives, while GWE pyrolyzed into 6 linear compounds and 10 aromatic ring products, and FGWE was found to produce only 3 linear compounds and 7 benzene ring derivatives. The differences originated from the contents of tannic acid and gallic acid. FGWE contained more gallic acid and little tannic acid, while TAS and GWE contained over 90% of tannin. Therefore, the results indicated that the LHS-1 strain could produce tannase to transform the tannic acid of GWE into gallic acid.

### 2.5. The Pyrolytic Products of TAS, GWE, and FGWE

**Figure 6:**
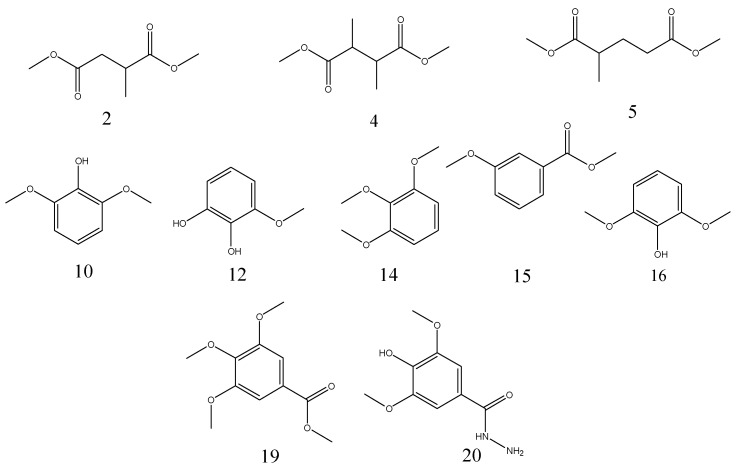
The chemical structures of the main pyrolytic products in TAS, GWE, and FGWE.

The pyrolytic products of TAS, GWE, and FGWE were composed of linear and benzene ring compounds. The Py-GC/MS signal peaks integrated the related abundances of individual compounds and determined the pyrolytic products of TAS, GWE, and FGTA, as shown in Table 3. It can be seen that the pyrolytic products of TAS had 14 compounds, in which 3-methoxy-benzene-1,2-diol was 20.77% of the highest content, and the content of 2,6-dimethoxyphenol, 1,2,3-trimethoxybenzene and 3,4-dimethoxyphenol were between 11.18% and 11.84%. GWE contained analogous linear and benzene ring pyrolytic products such as TAS. Sixteen pyrolytic products were appraised in GWE pyrolysis. Among them, 3,4,5-trimethoxybenzoic acid methyl ester and 3-methoxy-1,2-benzenediol represented 13.77% and 12.70%, respectively. Only four compounds represented between 5.32% and 8.22%. However, FGWE only produced pyrolytic products of 10 compounds. For FGWE, 3,4,5-trimethoxy-benzoic acid methyl ester represented 41.55% (the highest percentage), and 4-hydroxy-3,5-dimethoxy-benzoic acid hydrazide represented 7.42%. The pyrolysis products of benzene ring derivatives from FGWE were further identified as 3,4-dimethoxy-phenol, 1,2,3-trimethoxybenzene, 3-methoxy-benzoic acid methyl ester, 2,6-dimethoxy-phenol, 3,4-dimethoxy-benzoic acid methyl ester, 3,4,5-trimethoxybenzoic acid methyl ester, and 4-hydroxy-3,5-dimethoxy-benzoic acid hydrazide. The contents of main pyrolytic products in TAS, GWE, and FGWE are shown in Figure 6.

## 3. Discussion

Garnier et al. [8] reported temperatures effected on the pyrolytic process of hydrolysis–methylation of between 250 °C and 500 °C. When the temperature was above 500 °C, sufficient thermal energy probably led the formation of free radicals in the gas phase and caused thermal fragmentation reactions to occur. Therefore, we carried out pyrolytic processes with gallnut tannic acid at 380 °C for 5 s in order to control the radical fragmentations. All experiments were conducted in triplicate to study the reliability and stability, and the results provided enough evidence for good reproducibility of the method [10].

In general, Chinese gallnut contained 55~75% tannic acid, and 2~5% gallic acid and ellagic acid. GWE contained more than 90% of tannin [14]. Gallnut tannic acid is a β-glycoside bond polymer with one glucose core and five gallic acid esters. It is very easy for the LHS-1 strain or pyrolysis to break the ester bonds between the glucose core and gallic acid, like the benzoic acid and phenolic hydroxyl group. Those pyrolytic products with a benzene ring might be derived from splitting between the ester bonds in gallic acid and glucose, and the depside bonds among gallic acid, while liner pyrolytic products probably result because of the splitting among glucose.

Compared to TAS, GWE gave rise to linear and aromatic pyrolytic products of tannic acid as major products. It was obvious that both the varieties and quantities of linear products were reduced. Pyrolysis products like the ethyl-, dimethyl ester of butanedioic acid, dimethyl 2-methyladipate, and 1,2,6-trimethoxyhexane disappeared. While the aromatic products became more complex, new pyrolytic products, such as 3-methoxybenzoic acid methyl ester and 4-methoxy benzoic acid methyl ester, appeared. Benzoic acid 3,4,5-trimethoxybenzoic acid methyl ester had a greater presence, of about 13.77%. All these results might be caused due to the effects of water extraction, which decreased the degrees of polymerization of tannic acid, resulting in an increase in free gallic acid and polyphenols so the structures of methylate with the benzene ring were more complex. Besides, the other water-soluble impurities mixed in the Chinese gall medicinal materials could influence the pyrolytic products.

However, the pyrolysis products of FGWE were further simplified as compared with TAS and GWE. The total abundances of 3,4,5-trimethoxybenzoic acid methyl ester and 4-hydroxy-3,5-dimethoxy-benzoic acid hydrazide (pyrolysis and methylated products of gallic acid) were about 48.97%, which was more than 3.6 times greater than in GWE. The results might be because there was no tannic acid with a high polymerization degree in FGWE. The LHS-1 strain degraded most of tannic acid in GWE into gallic acid and small amounts of low molecular weight phenolic substances and glucose in FGWE. Then, glucose continued to be degraded as the carbon source and formed the linear pyrolysis products. Meanwhile, FGWE contained few other sources of mycelium except the metabolite of the LHS-1 strain. These results showed that the LHS-1 strain gave a high degradation rate of tannic acid, resulting in the formation of gallic acid.

From the above, the biodegradation mechanism of gallnut tannic acid was different from that of the Chinese gallnut aqueous extract. Obviously, the differences in the pyrolytic products that existed in the three samples originated from the compositions of tannic acid and gallic acid. The benzene ring or aromatic compounds came from the pyrolysis of the epside bonds of gallic acid. The main pyrolytic products of FGWE were 3,4,5-trimethoxybenzoic acid methyl ester, 3-methoxy-1,2-benzenediol, and 4-hydroxy-3,5-dimethoxy- benzoic acid hydrazide.

## 4. Materials and Methods

### 4.1. Raw Materials and Chemicals

Chinese gallnut powder (containing 56.8% total tannic acid) was provided as a raw material by the Nanjing Longyuan Natural Polyphenol Synthesis Factory (Nanjing, China) in China, and was kept at 4 °C before used. The other chemicals were purchased from commercial suppliers (Sigma-Aldrich, St. Louis, MO, USA; Merck, Darmstadt, Germany; laddin, Beijing, China). GWE, FGWE, and the LHS-1 strain were prepared by our lab.

### 4.2. Basal Culture Mediums

Plate sieve culture medium (tannic acid medium): The basal medium contained sucrose 20 g, NaNO_3_ 2.0 g, K_2_HPO_4_ 1.0 g, MgSO_4_·7H_2_O 0.5 g, KCl 0.5 g, FeSO_4_ 0.01 g, 1% brom ophenol blue, gallnut tannin 10 g, and agar 20 g in 1000 mL tap water.

Liquid fermentation medium: The basal medium contained sucrose 20 g, NaNO_3_ 2.0 g, K_2_HPO_4_ 1.0 g, MgSO_4_·7H_2_O 0.5 g, KCl 0.5 g, FeSO_4_ 0.01 g, and gallnut tannins 100 g in 1000 mL tap water. The above mediums were autoclaved at 121 °C for 20 min.

### 4.3. Microorganism and Culture Conditions

The LHS-1 strain was isolated from GWE in our laboratory previously, and was identified as a new variant of *A. niger* through traditional morphological identification and the phylogenetic tree of 18 s rDNA sequencing. It was then used for further study. The LHS-1 strain was kept for the culture of tannic acid agar slants stored at 4 °C and sub-cultured for regular intervals of four weeks. The cells were grown at 30 °C for 4 days shaking at 150 rpm.

### 4.4. The Preparation of GWE and FGWE Samples

Took 5 g of Chinese gallnut powder to mix with 50 mL distilled water in a 150-mL extractor at 50 °C for 1 h by vacuum cavitation extraction, then filtered and centrifuged. The filtrate was freeze-dried at −50 °C for 72 h to obtain GWE, and stored at 4 °C before use.

250 mL of GWE solution was then sterilized at 121 °C for 20 min. After being cooled to room temperature, the inoculated strain LHS-1 was added to the sterilized GWE solution, and the mixture was shaken at 150 rpm at 30 °C for 4 days. After the GWE was fermented by the LHS-1 strain, the fermented broth was filtered in a vacuum, and the filtrate was freeze-dried at −50 °C until the LHS-1 strain fermentation sample of GWE (FGWE) was obtained. The sample was then stored at 4 °C.

### 4.5. Analysis of Tannic Acid, Gallic Acid, and Tannin Enzyme Activities

Tannic acid was analyzed using Folin–Ciocalteau on the basis of the standard curve generated with tannic acid standard [9,10,11]. HPLC was used to assay tannic acid and gallic acid. The HPLC conditions were as follows: C18 (150 mm × *Φ* 4.6 mm × 5 μm) chromatographic column, PDA detector, methanol–water (0.5%) = 5:95 of mobile phase, 270-nm wavelength determination, 1 mL min^−1^ of flow speed.

The degradation rate of tannic acid was calculated with the formula: R=1−TAtTA0, where *R* is degradation rate of tannic acid, *TA*_0_ is the concentration of tannic acid in the TAA medium, and *TA_t_* is the concentration of tannic acid at the time of T(t).

A small amount of nutrient solution was taken, and the mycelium filtered as a crude enzyme liquid. Three tubes were marked as the blank, test, and control tubes, respectively. Gallic acid accumulation and tannase activities were assayed by the spectrophotometric method of methanolic rhodanine [15]. One unit (U) of tannase was defined as one micromole of gallic acid formed per minute. Tannin enzyme activities were determined according to the content of TA and GA.

### 4.6. The Box–Behnken Experiment on GWE Biodegradation

For determining the optimum parameters of GWE biodegradation with the LHS-1 strain, a Box–Behnken experiment was designed with three factors and three levels (Table 4). The parameters were studied with ranges as follows: temperature of 25~35 °C, initial pH of 4~6, and incubation period of 36~60 h.

### 4.7. Conditions of the Py-GC/MS Experiments

Py-GC/MS was performed using a double-shot pyrolyzer (Frontier Laboratories, model 2020i) attached to a GC/MS system Agilent 6890N (Santa Clara, CA, USA). TAS, GWE, and FGWE samples were analyzed by Py-GC/MS in accordance with procedures.

Samples of 0.4 mg of TAS, GWE, and FGWE were placed in their respective sample cups. Sufficient methylated derivatives were added with a microsyringe (TMAH, 25% methanol), and then the samples were placed in small crucible capsules and introduced into the furnace, which was preheated at 500 °C for 1 min. GC-MS analysis with the Agilent 6890 N system was performed. The GC was equipped with a low-to-mid polarity-fused silica capillary column of HP-5 of 30 m × 250 μm × 25 μm film thickness. Column temperature: initial temperature 50 °C for 5 min, with a subsequent increase rate of 10 °C/min until 280 °C, maintained for 15 min. The vaporizing chamber temperature was 300 °C and the carrier gas was helium. There was a constant pressure mode (6.0 kPa), and a split ratio of 20:1. Pyrolysis temperature was 380 °C for 5 s. The detector consisted of an Agilent 5975 mass selective detector and the electrical energy acquired was 70 eV. The compounds were identified by comparing their mass spectra with reference compounds from the NISTO_2_ and Wiley libraries. Traces corresponding to selected homologous series of chemical families were obtained by single ion monitoring (SIM) of characteristic ions.

## 5. Conclusions

TAS, GWE, and FGWE samples were analyzed using HPLC and Py-GC/MS. The optimization technology of the LHS-1 strain degrading GWE was determined by a response surface method. The optimum fermentation conditions of the LHS-1 strain were as follows: temperature of 31 °C, pH of 5, and a 50-h fermentation time. The tannase activity was 1.17 U·mL^−1^. Through comparing the mass spectra of analytes in NITS02 libraries and the retention times with the corresponding standard references, over 20 kinds of compounds were determined and annotated on the chromatogram. The pyrolytic products of all three samples were shown to be composed of linear and aromatic compounds. Among these, the total abundance of benzene ring derivatives of FGWE was about 48.97%. The metabolites of the LHS-1 strain contained almost no high polymerized tannic acid, giving a high degradation rate of tannic acid resulting in the formation of gallic acid and small amounts of phenolic compounds and glucose. Py-GC/MS is a convenient and efficient method for tracing tannic acid and gallic acid in fermentation broth, and has significant applicable value.

## Figures and Tables

**Table 1:** The Box–Behnken experimental design and corresponding tannase activity for the LHS-1 strain.

No.	*X*_1_ Temperature (°C)	*X*_2_ Initial pH	*X*_3_ Incubation Time (h)	*Y* Tannase Activity (U·mL^−1^)
1	−1	−1	0	0.892
2	1	−1	0	0.968
3	−1	1	0	0.944
4	1	1	0	1.016
5	−1	0	−1	0.852
6	1	0	−1	0.948
7	−1	0	1	0.936
8	1	0	1	1.004
9	0	−1	−1	0.944
10	0	1	−1	0.812
11	0	−1	1	0.924
12	0	1	1	0.972
13	0	0	0	1.164
14	0	0	0	1.14
15	0	0	0	1.192
16	0	0	0	1.22
17	0	0	0	1.204

**Table 2:** Variance analysis of items in the regression equation.

Sources of Variation	Sum of Squares	*df*	Mean Square	*F*-Value	*p*-Value
model	0.016	9	0.028	23.62	0.0002
*X*_1_	7.605 × 10^−5^	1	0.012	10.19	0.0152
*X*_2_	2.000 × 10^−6^	1	3.200 × 10^−5^	0.027	0.8746
*X*_3_	6.125 × 10^−4^	1	9.800 × 10^−3^	8.21	0.0242
*X*_1_*X*_2_	2.500 × 10^−7^	1	4.00 × 10^−6^	0.0033	0.9555
*X*_1_*X*_3_	1.225 × 10^−5^	1	1.960 × 10^−4^	0.16	0.6975
*X*_2_*X*_3_	5.063 × 10^−4^	1	8.100 × 10^−3^	6.78	0.0352
*X*_1_^2^	2.819 × 10^−3^	1	0.045	37.77	0.0005
*X*_2_^2^	4.145 × 10^−3^	1	0.066	55.53	0.0001
*X*_3_^2^	5.571 × 10^−3^	1	0.089	74.64	<0.0001
residual	5.225 × 10^−4^	7	1.194 × 10^−3^		
lack-of-fit item	2.665 × 10^−4^	3	1.421 × 10^−3^	1.39	0.3680
pure error	2.560 × 10^−4^	4	1.024 × 10^−3^		
total	0.016	16			

**Table 3:** The relative intensities of pyrolytic products of TAS, GWE, and FGWE.

Peak	Retention Time (min)	MW	Formula	Compound	Sample Total Peak Area, %		
					TAS	GWE	FGWE
1	9.946	146	C_6_H_10_O_4_	Succinic acid, dimethyl ester	1.68	0.62	-
2	10.656	160	C_7_H_12_O_4_	Succinic acid, methyl-, dimethyl ester	8.11	1.8	2.57
3	11.182	124	C_7_H_8_O_2_	Phenol, 2-methoxy-	2.17	1.37	-
4	11.348	174	C_8_H_14_O_4_	Dimethyl 2,3-dimethylsuccinate	6.33	0.74	2.69
5	11.520	158	C_7_H_10_O_4_	Dimethyl ethylidene malonate	1.89	0.67	-
6	11.749	174	C_8_H_14_O_4_	Pentane dioic acid, 2-methyl-, dimethyl ester	5.35	0.71	2.62
7	12.184	174	C_8_H_14_O_4_	Butane dioic acid, ethyl-, dimethyl ester	1.30	-	-
8	12.584	188	C_9_H_16_O_4_	Dimethyl 2-methyladipate	0.73	-	-
9	12.784	176	C_9_H_20_O_3_	1,2,6-Trimethoxy-hexane	1.86	-	-
10	13.963	154	C_8_H_10_O_3_	Phenol, 3,4-dimethoxy-	11.84	6.22	0.92
11	14.009	152	C_9_H_12_O_2_	3,5-Dimethoxytoluene	-	0.81	-
12	14.512	140	C_7_H_8_O_3_	1,2-Benzenediol, 3-methoxy-	20.77	12.70	-
13	14.844	140	C_7_H_8_O_3_	2-Methoxyresorcinol	-	1.62	-
14	14.964	168	C_9_H_12_O_3_	1,2,3-Trimethoxybenzene	11.18	7.63	4.39
15	15.439	166	C_9_H_10_O_3_	3-Methoxybenzoic acid methyl ester	-	5.32	1.81
16	15.640	154	C_8_H_10_O_3_	Phenol, 2,6-dimethoxy-	11.24	8.22	0.71
17	15.937	166	C_9_H_10_O_3_	Methyl 4-methoxybenzoate	-	0.91	-
18	18.793	196	C_10_H_12_O_4_	Benzoic acid, 3,4-dimethoxy-, methyl ester	-	1.02	1.12
19	20.383	226	C_11_H_14_O_5_	Benzoic acid, 3,4,5-trimethoxy-, methyl ester	3.57	13.77	41.55
20	21.150	212	C_9_H_12_N_2_O_4_	Benzoic acid, 4-hydroxy-3,5-dimethoxy-, hydrazide	-	-	7.42

Note: “-” indicates a compound peak area of <0.6% in the table.

**Table 4:** Factors and levels in response surface analysis.

Factors	Code	Levels		
		−1	0	1
A: temperature (°C)	*X*_1_	25	30	35
B: initial pH	*X*_2_	4	5	6
C: incubation period (h)	*X*_3_	36	48	60
